# Optimizing the Endometrial Factor in Recurrent Implantation Failure: From Established Diagnostics to Mechanism-Informed Management

**DOI:** 10.3390/jcm15187039

**Published:** 2026-09-11

**Authors:** Athanasios Zikopoulos, Efthalia Moustakli, Anastasios Potiris, Vasilios Sebastian Paraschos, Ariadni Theodora Katopodi, Nikolaos Kathopoulis, Ismini Anagnostaki, Ioannis Tsakiridis, Themistoklis Dagklis, Eriketi Kokkosi, Angeliki Sarella, Konstantinos Zikopoulos, Sofoklis Stavros

**Affiliations:** 1 Department of Reproductive Medicine and Surgery, University College London Hospitals NHS Foundation Trust, 235 Euston Road, London NW1 2BU, UK; 2 Department of Nursing, School of Health Sciences, University of Ioannina, 45500 Ioannina, Greece; ef.moustakli@uoi.gr; 3 Third Department of Obstetrics and Gynecology, University General Hospital “ATTIKON”, Medical School, National and Kapodistrian University of Athens, 12462 Athens, Greece; apotiris@med.uoa.gr (A.P.); sfstavrou@med.uoa.gr (S.S.); 4 Department of Obstetrics and Gynecology, Corewell Health Hospital, 4700 Schaefer Road Suite 310, Dearborn, MI 48126, USA; vasilispar10@gmail.com; 5 Medical School, Aristotle University of Thessaloniki, 54124 Thessaloniki, Greece; katopodi.ariadni@gmail.com; 6 Medical School, National and Kapodistrian University of Athens, 11528 Athens, Greece; isanagnostaki3@gmail.com; 7 Third Department of Obstetrics and Gynecology, General Hospital Ippokratio, Medical School, Aristotle University of Thessaloniki, 54642 Thessaloniki, Greece; iotsakir@gmail.com (I.T.); tdagklis@gmail.com (T.D.); 8 Department of Midwifery, Faculty of Health and Caring Sciences, University of West Attica, 12243 Athens, Greece; ekokkosi@uniwa.gr (E.K.); asare@uniwa.gr (A.S.); 9 Department of Obstetrics and Gynecology, University Hospital of Ioannina, 45500 Ioannina, Greece

**Keywords:** recurrent implantation failure, endometrial receptivity, window of implantation, progesterone resistance, decidualization, chronic endometritis, uterine microbiome, uterine natural killer cells, personalized embryo transfer, assisted reproductive technology

## Abstract

**Background:** Recurrent implantation failure (RIF) remains one of the most challenging presentations in assisted reproductive technology and is characterized by failure to achieve clinical pregnancy despite the transfer of multiple good-quality embryos. Advances in embryo selection and preimplantation genetic testing have highlighted the contribution of endometrial factors to a substantial proportion of otherwise unexplained implantation failures. **Methods**: This narrative review summarizes current evidence regarding endometrial receptivity, the principal endometrial mechanisms implicated in RIF, contemporary diagnostic approaches, and available therapeutic strategies. Electronic literature searches were conducted using PubMed/MEDLINE, Scopus, ScienceDirect, and the Cochrane Library for publications available up to June 2026. **Results**: Anatomical, inflammatory, temporal, hormonal, immunological, and vascular endometrial causes of RIF can all be broadly categorized. Progesterone resistance, impaired decidualization, chronic endometritis, microbiome dysbiosis, displacement of the window of implantation, and immune dysregulation represent proposed mechanisms contributing to implantation failure in selected patients. Diagnostic evaluation is most effective when performed using a stepwise approach that prioritizes clinically actionable findings and avoids indiscriminate testing. Management primarily focuses on optimizing hormonal support, whereas immunomodulatory and antithrombotic therapies should be reserved for carefully selected patients. Emerging approaches, including granulocyte colony-stimulating factor, platelet-rich plasma, and microbiome-directed therapies, have shown promising preliminary results in selected patient populations; however, further validation is required. **Conclusions**: RIF is best regarded as a heterogeneous clinical outcome rather than a single disease entity, reflecting multiple disturbances in endometrial receptivity. Overall, current evidence supports a mechanism-informed approach that integrates targeted diagnostics with individualized, evidence-based therapeutic strategies.

## 1. Introduction

Recurrent implantation failure (RIF) remains one of the most conceptually challenging conditions in reproductive medicine. It is not a disease in its own right but rather a clinical condition characterized by failure to achieve implantation despite repeated embryo transfers involving embryos with favorable developmental, morphological, and, when available, genetic characteristics. Its management requires balancing the risks of under-investigation, which may contribute to further failed cycles, against those of over-investigation, which may expose patients to unnecessary procedures and costs [1,2].

The lack of a universally agreed-upon definition has further complicated the concept of RIF. Earlier definitions required at least three failed embryo transfer cycles or the transfer of at least 10 good-quality embryos. However, these parameters were formulated during a time when embryo transfer at the cleavage stage was common practice, and genetic screening of embryos was not routinely performed [1,3]. Advances in reproductive medicine, including blastocyst culture, single-embryo transfer, cryopreservation, and preimplantation genetic testing for aneuploidy (PGT-A), have reduced the relevance of rigid numerical cut-offs for defining RIF. Thus, the 2023 ESHRE good practice recommendations suggested that RIF should be considered on an individualized basis according to the calculated cumulative chance of implantation, taking into account factors including maternal age, embryo stage and quality, number of previous embryo transfers, and embryo ploidy where applicable [4]. Accordingly, throughout this review, RIF is operationally considered in accordance with the 2023 ESHRE good practice recommendations, whereby further investigation is considered when the cumulative predicted chance of implantation has exceeded 60% without achieving implantation, rather than after a predetermined number of failed embryo transfers [4].

Experience with euploid embryo transfers has further emphasized the importance of considering factors beyond traditional morphological assessment of the embryo [5,6]. Persistent implantation failure following the transfer of euploid embryos under apparently optimal laboratory and embryo transfer conditions reduces the likelihood that the embryo is the sole cause of implantation failure. This shifts the focus toward maternal factors, including the uterine cavity, endometrial receptivity, and embryo–endometrium interactions [7,8]. However, even euploidy does not guarantee implantation competence, and RIF cannot be automatically attributed to endometrial dysfunction. Current evidence suggests that endometrial abnormalities contribute to implantation failure in a subset of patients [8,9].

The endometrium is a highly dynamic tissue whose receptivity depends on the coordinated interplay of hormonal signaling, decidualization, immune adaptation, vascular remodeling, and molecular crosstalk with the embryo [10,11]. Disruption of any of these processes may impair embryo implantation, even when the embryo is developmentally competent. Conversely, the growing number of diagnostic tests and therapeutic interventions has created uncertainty regarding which approaches provide meaningful clinical benefit and which do not [12,13].

The biological heterogeneity of RIF poses an additional challenge. Clinical trials often include heterogeneous patient populations with diverse underlying biological mechanisms. This heterogeneity may influence trial outcomes and contribute to inconsistent or non-reproducible findings. This issue is well exemplified by endometrial scratching: encouraging initial findings were not confirmed in randomized controlled trials [14,15]. Consequently, a major clinical challenge is identifying the subgroup of patients with RIF most likely to benefit from a given intervention.

To date, most reviews and clinical recommendations have addressed individual diagnostic tests, endometrial abnormalities, or adjunctive therapies separately, with limited integration into a mechanism-informed framework. Similarly, limited attention has been paid to linking the fundamental biology of the endometrium with the clinical utility of diagnostic approaches and the evidence supporting therapeutic interventions. For this reason, this narrative review provides an overview of the endometrial factors involved in RIF within an integrated, mechanism-informed framework. The review describes the physiological basis of endometrial receptivity, outlines key endometrial mechanisms involved in implantation failure, assesses current diagnostic tools, and critically appraises established, investigational, and controversial therapeutic approaches.

## 2. Literature Search Strategy

This narrative review was conducted to provide a clinically oriented synthesis of the endometrial factors implicated in recurrent implantation failure (RIF), with particular emphasis on the mechanisms governing endometrial receptivity, their diagnostic evaluation, and corresponding therapeutic interventions. Electronic literature searches were conducted using PubMed/MEDLINE, Scopus, ScienceDirect, and the Cochrane Library for publications available up to June 2026. The search strategy combined the terms “recurrent implantation failure” and “implantation failure” with terms related to endometrial physiology and dysfunction, including “endometrial receptivity,” “window of implantation,” “decidualization,” “progesterone resistance,” “chronic endometritis,” “endometrial microbiome,” “uterine natural killer cells,” “immune dysregulation,” “vascular dysfunction,” and “thrombophilia.” Additional searches incorporated relevant diagnostic and therapeutic terms, including “endometrial receptivity testing,” “personalized embryo transfer,” “progesterone supplementation,” “corticosteroids,” “aspirin,” “low-molecular-weight heparin,” “platelet-rich plasma,” “granulocyte colony-stimulating factor,” “human chorionic gonadotropin,” “hCG,” “endometrial scratching,” “intralipid,” and “intravenous immunoglobulin.” The complete database-specific search strategies are provided in Table A1.

Publications were considered eligible if they addressed endometrial mechanisms, diagnostic approaches, or therapeutic interventions relevant to RIF or repeated failure of embryo implantation. Clinical practice guidelines, randomized controlled trials, systematic reviews, meta-analyses, prospective and retrospective observational studies, and clinically relevant mechanistic investigations were considered. Studies focusing exclusively on embryonic or paternal factors without assessment of the endometrial contribution to implantation failure, isolated case reports, conference abstracts lacking sufficient methodological information, and publications not available in English were excluded. Preclinical studies were considered selectively when they provided mechanistic context for endometrial pathways for which human evidence remains limited. Reference lists of relevant primary studies, reviews, and clinical guidance documents were manually examined to identify additional pertinent publications.

As this article was designed as a narrative rather than a systematic review, records were not processed through a formal systematic-review screening workflow. Consequently, no PRISMA-based study-selection process, formal duplicate-removal procedure, or formal risk-of-bias assessment was undertaken. The retrieval counts reported in Table A1 therefore represent the results returned by each search and should not be interpreted as numbers of unique screened records. Formal screening and exclusion numbers are consequently not reported.

Evidence was critically evaluated using a narrative evidence-weighting approach based on study design, methodological quality, relevance to the RIF population, sample size, presence of an appropriate comparator, consistency with the wider evidence base, and clinical applicability. Greater weight was assigned to clinical practice guidelines, randomized controlled trials, systematic reviews, and meta-analyses than to small observational or uncontrolled studies, particularly when findings were conflicting. Particular emphasis was placed on clinically meaningful reproductive outcomes, especially live birth and ongoing pregnancy, rather than surrogate outcomes alone. Evidence from small, uncontrolled, or mechanistic studies was used primarily to provide biological context or to characterize emerging and investigational approaches. This approach was used to distinguish established clinical practices from interventions for which evidence remains limited, inconsistent, or experimental.

## 3. Physiology of Endometrial Receptivity and the Window of Implantation

Coordinated apposition, attachment, and invasion of a developmentally competent blastocyst into a well-prepared endometrium are necessary for successful implantation [13]. Therefore, endometrial receptivity is a transient, tightly regulated physiological state resulting from the coordinated interplay of hormonal, molecular, cellular, immunological, and vascular processes rather than a static characteristic. When components of this receptive program are disrupted, many of the diagnostic and therapeutic strategies discussed later in this review aim to identify and restore these abnormalities [10,16,17].

### 3.1. Hormonal Regulation and Decidualization

The structural basis needed for implantation is established during the proliferative phase. The endometrium experiences glandular growth, vascular remodeling, and epithelial and stromal proliferation in response to estradiol, which primarily acts through estrogen receptor-α [18,19]. Angiogenesis and extracellular matrix remodeling are facilitated by matrix metalloproteinases, vascular endothelial growth factor (VEGF), and other locally generated mediators [20,21]. Despite being a common clinical indicator of endometrial growth, endometrial thickness is not a perfect proxy for functional receptivity. There is no single criterion that can accurately differentiate between receptive and non-receptive endometrium; however, a thin endometrium, typically described as a thickness below approximately 7 mm, has been linked to reduced implantation and pregnancy rates [22,23].

After ovulation, progesterone induces the transition from the proliferative to the secretory phase by activating the progesterone receptor isoforms PR-A and PR-B [24]. During the window of implantation, stromal fibroblasts undergo decidualization, a crucial process for the establishment and maintenance of endometrial receptivity, while the endometrial glands acquire a secretory phenotype. Decidualization is accompanied by extensive morphological, transcriptional, metabolic, and secretory changes, including the expression of prolactin, insulin-like growth factor-binding protein 1 (IGFBP-1), tissue factor, and numerous cytokines and growth factors. Interconnected transcriptional pathways, including FOXO1, HOXA10, HOXA11, and HAND2, regulate these alterations [25,26,27,28]. Decidualized stromal cells not only support embryo attachment but also control trophoblast invasion, support maternal immunological tolerance, and create the vascular and metabolic conditions necessary for early embryonic development [29].

### 3.2. The Window of Implantation

The window of implantation (WOI) is the brief period during which the endometrium develops the cellular and molecular characteristics required to facilitate blastocyst attachment. This interval typically occurs during the mid-luteal phase of a normal 28-day menstrual cycle, approximately between cycle days 20 and 24 [30,31]. In programmed frozen embryo transfer cycles, blastocyst transfer is typically scheduled after approximately 5 days of progesterone exposure. However, the timing and duration of endometrial receptivity may vary according to the endometrial preparation protocol and interindividual biological variability [32,33,34,35,36].

Coordinated alterations in adhesion molecules, cytokines, growth factors, and transcriptional regulators, such as integrins, leukemia inhibitory factor (LIF), HOXA10, and HOXA11, occur in the endometrium during this time [37,38,39,40,41]. Transcriptomic studies have identified distinct molecular profiles corresponding to the pre-receptive, receptive, and post-receptive phases of the endometrium. However, although these molecular states can be identified, current evidence does not support the routine use of transcriptomic testing or personalized embryo transfer to improve reproductive outcomes [35,42].

### 3.3. Embryo–Endometrium Communication

A dynamic bidirectional interaction between the embryo and the endometrium is essential for implantation. Human chorionic gonadotropin (hCG) and interleukin-1β (IL-1β) are key signaling molecules secreted by the blastocyst that may regulate tissue remodeling, angiogenesis, immune modulation, and endometrial receptivity [10,30]. On the other hand, cytokines, chemokines, growth factors, extracellular vesicles, and lipid mediators that regulate trophoblast invasion and embryo adhesion are produced by endometrial epithelial and stromal cells. Implantation failure may occur as a result of disrupted molecular crosstalk between the embryo and the endometrium, even when both appear normal on individual assessment [43,44].

### 3.4. Immune and Vascular Adaptation

Immune and vascular adaptations are additional components of the receptive endometrial phenotype. Uterine natural killer (uNK) cell numbers increase during the secretory phase of the menstrual cycle and early pregnancy, where they regulate trophoblast function, spiral artery remodeling, cytokine production, and local immune homeostasis. Unlike peripheral NK cells, uNK cells have relatively low cytotoxicity and are more involved in regulatory and tissue-remodeling processes [45,46,47,48]. Although alterations in uNK cell number, phenotype, and function have been reported in women with RIF, the available evidence is insufficient to support their use as diagnostic biomarkers [49].

For implantation and early placental development, adequate angiogenesis and endometrial perfusion are equally necessary. However, in the absence of a clear clinical justification, the physiological significance of vascular adaptation should not be used to justify empirical antithrombotic therapy [50].

Hormonal signaling, decidualization, immunological adaptability, vascular remodeling, and embryo–endometrium communication must all be successfully integrated for implantation to occur. Implantation failure may result from impaired endometrial receptivity caused by disruption of one or more of these interrelated processes [1,10,51]. While acknowledging that the existence of a physiologically plausible abnormality does not necessarily establish its causal role or therapeutic actionability, this physiological paradigm serves as the foundation for categorizing the possible endometrial processes implicated in RIF [52,53,54].

## 4. Mechanistic Classification of Endometrial Causes of RIF

The biological heterogeneity of recurrent implantation failure cannot be adequately captured by a simple dichotomy of successful versus failed implantation. Patients with recurrent implantation failure may exhibit diverse underlying biological abnormalities that frequently coexist and interact [1,55,56]. Conceptually, endometrial factors can be classified into six categories: anatomical, inflammatory/infectious, temporal, hormonal, immunological, and vascular/thrombotic. It is important to note that these factors do not represent proven causes in all patients. However, they represent a biologically sensible way to approach these issues [1,57,58,59].

### 4.1. Anatomical Factors

Structural abnormalities of the uterine cavity may impair implantation by disrupting the receptive endometrial surface, distorting the intrauterine environment, altering local perfusion, and promoting inflammatory and fibrotic changes. Submucosal fibroids, endometrial polyps, intrauterine adhesions, and congenital uterine malformations, especially septate or subseptate uteri, are the main abnormalities. Despite their distinct pathophysiological mechanisms and clinical implications, these disorders may impair embryo apposition, attachment, or subsequent trophoblast invasion [60,61].

The clinical impact of a structural abnormality depends on its type, size, and location. While the clinical significance of small polyps or non-cavity-distorting intramural fibroids is less well established, lesions that distort the uterine cavity have a greater potential to impair implantation [62,63,64]. Adenomyosis is not an intracavitary lesion, but it can hinder implantation through decreased decidualization, altered uterine contractility, persistent inflammation, and abnormal progesterone signaling. Therefore, anatomical findings should be evaluated according to their potential impact on the implantation environment rather than their presence alone [65,66,67,68].

### 4.2. Inflammatory and Infectious Factors

Chronic endometritis is characterized by chronic, typically subclinical inflammation of the endometrium and is often asymptomatic. The reported prevalence of chronic endometritis in patients with recurrent implantation failure varies widely, ranging from approximately 7.7% to 63.8% across published studies, largely because of differences in patient populations, biopsy timing, histopathological criteria, immunohistochemical markers, and the threshold used to define endometrial plasma cell infiltration [69,70]. In particular, differences in plasma-cell thresholds and the use of CD138 or MUM-1 immunohistochemistry contribute substantially to variability in reported prevalence. Chronic inflammation can influence endometrial gene expression, decidualization, and the immune milieu necessary for implantation. However, the causal role of chronic endometritis in RIF and the effect of its treatment on live birth rates remain poorly understood [71,72].

The potential role of the endometrial microbiota has also attracted considerable interest. The conventional belief that the uterine cavity is consistently sterile has been called into question by the detection of microbial signals in samples taken from the upper reproductive tract using molecular sequencing techniques. However, because the endometrium is a low-biomass environment, results are particularly susceptible to contamination from the vagina, cervix, sampling tools, laboratory reagents, and sequencing procedures [73,74,75]. Some studies have associated *Lactobacillus*-dominant microbial profiles with improved reproductive outcomes, whereas non-*Lactobacillus*-dominant profiles have been associated with implantation failure. However, causal interpretation and therapeutic applicability are currently limited by methodological variability and the lack of established criteria for endometrial dysbiosis [76,77].

Taken together, current evidence supports a distinction between chronic endometritis and endometrial microbiome alterations in terms of clinical actionability. Chronic endometritis represents a potentially treatable endometrial abnormality when supported by appropriate histological evidence, although its causal contribution to RIF and the effect of treatment on live birth remain uncertain. In contrast, endometrial microbiome profiling remains primarily investigational, and the detection of altered microbial signatures alone should not currently be interpreted as a treatable cause of RIF.

### 4.3. Temporal Asynchrony and Window-of-Implantation Displacement

Temporal synchronization between endometrial maturation and embryonic development is necessary for successful implantation. Theoretically, embryo transfer might occur during a pre-receptive or post-receptive endometrial state if there is a mismatch between the developmental stage of the embryo and endometrial receptivity. The biological plausibility of temporal asynchrony as a contributor to implantation failure is supported by transcriptomic studies demonstrating molecular profiles corresponding to different stages of endometrial maturation [78,79,80,81].

Several studies have reported displacement of the expected window of implantation (WOI) in subsets of women with RIF. A systematic review and meta-analysis estimated the prevalence of WOI displacement based on endometrial receptivity analysis at 34% (95% CI 24–43%) in RIF/poor-prognosis populations, although substantial between-study heterogeneity was observed (I^2^ = 87.9%) [82]. However, considerable uncertainty remains regarding the true prevalence, reproducibility across menstrual cycles, and clinical significance of this finding, particularly given the heterogeneity of RIF populations and the definitions of RIF used across studies, as well as the inconsistent evidence supporting routine use of endometrial receptivity testing [42,82,83]. Importantly, the identification of a non-receptive transcriptomic profile does not establish that previous implantation failures resulted from temporal displacement or that adjustment of embryo-transfer timing will improve live birth rates. Therefore, WOI displacement currently has greater value as a mechanistic hypothesis than as an established clinical diagnosis or basis for personalized embryo transfer in RIF [84,85,86].

### 4.4. Hormonal Dysfunction, Progesterone Resistance, and Decidualization Failure

The development of endometrial receptivity depends critically on progesterone-dependent decidualization. Inadequate progesterone exposure, variability in progesterone absorption or metabolism, improper timing of supplementation, or reduced cellular responsiveness despite adequate hormone concentrations can disrupt this process. These conditions are clinically distinct: true progesterone resistance remains difficult to identify in routine practice, whereas inadequate progesterone exposure can be identified by serum progesterone assessment and corrected by optimizing luteal phase support [28,87,88].

Altered progesterone-receptor expression or signaling, dysregulation of receptor co-regulators, epigenetic modification of progesterone-responsive genes, and disruption of decidualization pathways involving FOXO1, HOXA10, HOXA11, and HAND2 are some of the hypothesized mechanisms of progesterone resistance. Although progesterone resistance can exist in the absence of clinically evident illness, it has been most thoroughly described in endometriosis and adenomyosis. However, there is still no validated clinical test that can accurately identify this phenotype or predict its response to a specific intervention [40,89,90]. Accordingly, inadequate progesterone exposure and true endometrial progesterone resistance should not be considered interchangeable explanations for RIF: the former is clinically measurable and potentially correctable, whereas the latter remains primarily a mechanistic concept without a validated diagnostic or targeted therapeutic strategy.

### 4.5. Immunological Factors

The endometrial immune environment must support implantation, tissue remodeling, and the establishment of maternal immune tolerance to the semi-allogeneic conceptus. uNK cells are the best-characterized immune cell population in the endometrium and play essential roles in cytokine production, trophoblast regulation, and spiral artery remodeling. Some studies have reported alterations in uNK cell number, phenotype, and function in women with RIF; however, these findings have not been consistently replicated [1,49,91,92,93].

Other proposed mechanisms include an imbalance between pro-inflammatory and regulatory immune responses, alterations in the function of Th1, Th2, Th17, and regulatory T cells, and abnormalities in cytokine and chemokine signaling pathways. Autoimmune diseases may also affect early reproductive processes, although their role in implantation failure should be distinguished from their association with pregnancy loss, which has been more extensively studied [44,94]. Substantial variability remains in immune testing methods, sampling and analysis, timing of testing, and the criteria used to define immune dysregulation. Consequently, current evidence supports an immunological contribution to implantation biology but does not support routine immune testing or empirical immunomodulatory therapy in RIF in the absence of a validated biomarker or established clinical indication [95,96].

### 4.6. Vascular and Thrombotic Factors

Important elements of implantation and early placental development include angiogenesis, vascular remodeling, and sufficient endometrial perfusion. Impaired subendometrial perfusion, abnormal angiogenic signaling, aberrant spiral artery remodeling, complement activation, and microvascular dysfunction are among the suggested vascular causes of implantation failure. However, the ability of uterine or subendometrial blood flow measurements to predict implantation outcomes or guide treatment remains uncertain [97,98,99].

Antiphospholipid syndrome is the acquired thrombophilic condition with the most well-established adverse effects on reproduction. However, it appears to be more strongly associated with recurrent pregnancy loss and subsequent placental complications than with implantation failure [100,101]. Routine thrombophilia screening is not recommended in patients with RIF in the absence of a relevant personal or family history, and the evidence linking inherited thrombophilias to RIF remains inconsistent. Similarly, although adequate endometrial perfusion is physiologically important, vascular or thrombotic mechanisms should not be assumed solely on the basis of implantation failure, and current evidence does not support the empirical use of aspirin or low-molecular-weight heparin in unselected patients with RIF [102,103].

Although presented individually, these processes often coexist and interact. Chronic inflammation may impair progesterone responsiveness, hormonal dysfunction may alter immunological regulation, and immune or vascular abnormalities may disrupt decidualization and embryo–endometrium communication [10,28,104]. Anatomical abnormalities such as adenomyosis may similarly exert hormonal, inflammatory, vascular, and mechanical effects. Therefore, RIF should not be regarded as a single endometrial pathological entity but rather as a heterogeneous clinical outcome resulting from multiple converging abnormalities [66,105].

The principal mechanistic categories, corresponding diagnostic approaches, and current levels of clinical actionability are summarized in Table 1.

## 5. Diagnostic Workup

Diagnostic evaluation of recurrent implantation failure should prioritize abnormalities that are clinically relevant, reproducible, and potentially actionable. Repeated treatment failure does not always imply endometrial disease, but persistent implantation failure may need a more thorough evaluation than that performed prior to a first IVF round. Incidental or poorly verified results from indiscriminate testing might raise costs and promote unsupported treatments [1,55,106]. Conversely, an overly limited evaluation may fail to identify clinically relevant uterine, hormonal, or procedural abnormalities. Therefore, a sequential diagnostic approach should be adopted, beginning with established investigations to confirm the RIF phenotype before proceeding to selective or investigational testing [2,107].

### 5.1. Reframing the Diagnostic Question

Before conducting any endometrial studies, it would be best for healthcare practitioners to first ensure that the patient’s prior treatment record is consistent with a reasonable likelihood of implantation problems. Current guidelines recommend an individualized treatment approach that incorporates maternal age, ovarian response, embryo stage and quality, the number of previous embryo transfers, and embryo ploidy status, when available [4,108].

Embryonic, laboratory, and procedural issues should be reconsidered. This includes embryonic development and morphology, available embryo ploidy information, the limitations of PGT-A, laboratory-related factors, post-thaw embryo survival, transfer difficulty, catheter placement, uterine contractility, the endometrial preparation protocol, and the timing of embryo transfer relative to progesterone administration [32]. Clinical factors should also be taken into consideration. These include BMI, smoking, thyroid disorder, metabolic disorder that is poorly managed, and male factors in particular. A comprehensive endometrial evaluation should be undertaken only after potentially correctable non-endometrial factors have been identified and addressed [109,110]. Notably, RIF does not itself establish an endometrial cause, and indiscriminate endometrial testing may identify abnormalities of uncertain causal or clinical significance.

### 5.2. Established and Clinically Indicated Investigations

Evaluation of uterine anatomy represents a key component of the diagnostic workup for RIF [111]. Transvaginal ultrasound is the preferred first-line imaging modality and can identify uterine fibroids, endometrial polyps, adenomyosis, congenital uterine anomalies, and abnormalities of endometrial development. Three-dimensional (3D) transvaginal ultrasound or saline infusion sonography may provide additional diagnostic information when an intracavitary lesion or congenital uterine anomaly is suspected [112,113,114]. Hysteroscopy enables direct visualization of the uterine cavity while allowing simultaneous treatment of clinically significant abnormalities, including endometrial polyps, intrauterine adhesions, uterine septa, and submucosal fibroids. However, the routine use of hysteroscopy in all women with RIF who have normal findings on high-quality transvaginal ultrasound remains controversial [115].

Cycle preparation and luteal phase support also need to be carefully evaluated. Important considerations include endometrial maturation, the schedule of estrogen administration for programmed cycles, the timing and route of progesterone supplementation, and treatment adherence [116,117,118]. Measurement of serum progesterone levels may be useful in programmed frozen embryo transfer cycles because interindividual variability in progesterone absorption and metabolism can result in inadequate circulating progesterone concentrations despite appropriate supplementation. However, the optimal timing of measurement and serum progesterone threshold remain protocol-specific, and a single serum measurement does not reflect endometrial sensitivity to progesterone [119,120,121].

Testing for chronic endometritis may be considered in selected cases of recurrent unexplained implantation failure, especially when clinical history, ultrasound findings, hysteroscopic findings, or reproductive history raise suspicion [122]. Endometrial biopsy, combined with histological examination and CD138 immunohistochemistry, is one of the most widely used methods for detecting endometrial stromal plasma cells. Variability in the methods and diagnostic criteria used for chronic endometritis makes comparison across studies difficult [123,124]. Thus, chronic endometritis testing is best interpreted in the context of the overall clinical picture rather than as a routine investigation for all patients with RIF.

### 5.3. Selected and Investigational Assessments

Endometrial transcriptomic receptivity tests, such as the Endometrial Receptivity Analysis and others, seek to categorize the endometrium as pre-receptive, receptive, and post-receptive. Although these tests can distinguish different endometrial gene expression profiles, randomized evidence has not demonstrated that test-guided management improves live birth rates. In a multicenter randomized clinical trial of women undergoing single euploid frozen embryo transfer, which excluded patients with RIF, live birth occurred in 58.5% (223/381) of women undergoing receptivity-timed transfer compared with 61.9% (239/386) undergoing standard-timed transfer (RR 0.95, 95% CI 0.79–1.13; *p* = 0.38). Consequently, current evidence does not support the routine clinical use of transcriptomic receptivity testing [35]. If testing is considered after repeated failures of euploid embryo transfer and exclusion of known causes, patients should be informed of its uncertain clinical value [125].

Assessment of the endometrial and reproductive tract microbiota remains investigational. Current methodologies vary with respect to sampling site, sampling technique, sequencing platform, bioinformatic analysis, and the criteria used to define dysbiosis [126,127]. Moreover, the low microbial biomass of the endometrium, together with the risk of contamination from the lower reproductive tract, sampling devices, laboratory reagents, and the environment, further complicates interpretation of the findings. Therefore, in the absence of established diagnostic and therapeutic thresholds, microbiome analysis cannot be routinely recommended for RIF [76,127].

Tests for thrombophilia and autoimmune disorders should be guided by the individual’s personal or family medical history rather than performed indiscriminately. A history of thromboembolic disease, recurrent miscarriages, placental complications, or clinical findings suggestive of an autoimmune condition or antiphospholipid syndrome would provide grounds for testing. There is no scientific basis for thrombophilia testing solely on the basis of RIF [103,128].

Immunological assessment remains one of the most controversial aspects of RIF evaluation. Peripheral blood NK cell counts do not reflect the uterine immune microenvironment and should not be used as surrogate markers of endometrial immune status. Determination of endometrial uNK-cell levels, cytokine assays, T-helper cell balance (Th1/Th2), HLA matching, and other immune assessments are not standardized, lack validated reference ranges, and have yet to demonstrate their ability to identify patients who would benefit from immunotherapy [4,129].

The use of Doppler analysis of the uterine and subendometrial vessels has also been suggested as a method for assessing endometrial perfusion. Although some evidence suggests an association between altered vascular indices and poor reproductive outcomes, the assessment methods and diagnostic criteria remain poorly standardized. Moreover, Doppler assessment has not been shown to improve clinical management or reproductive outcomes and should therefore not be used routinely to justify empirical therapy [130,131].

### 5.4. Integrating Diagnostic Findings

The aim of diagnostic testing is not merely to identify abnormalities but to determine whether they are reproducible, biologically plausible, and linked to interventions with proven clinical benefit. An abnormal test result does not necessarily identify the cause of previous implantation failures [132].

The presence of multiple abnormalities should not justify unnecessary additional testing or the indiscriminate use of multiple adjunctive therapies. Instead, findings should be interpreted according to the strength of the evidence linking them to implantation failure and whether they can guide interventions that improve clinically meaningful reproductive outcomes. When novel or additional tests are considered, their interpretation, potential therapeutic implications, costs, and risks should be clearly communicated to patients [4,133].

The proposed stepwise diagnostic framework and the current clinical status of the available investigations are summarized in Table 2.

### 5.5. Common Diagnostic Pitfalls

The evaluation of RIF is complicated by several recurring challenges. The first is initiating comprehensive endometrial testing before confirming that previous treatment failures cannot be explained by laboratory, hormonal, embryonic, or embryo transfer-related factors. Another is interpreting the identification of an abnormality as evidence of causation, especially when the test has no established clinical utility, validated thresholds, or standardized methodology [1].

Other challenges include performing extensive thrombophilia or immunological testing without a clear therapeutic indication, using peripheral biomarkers as surrogates for the uterine microenvironment, and extrapolating research findings to justify empirical or combination adjunctive therapies. Conversely, when a patient’s history suggests a multifactorial process, identifying one potentially significant abnormality should not immediately rule out other clinically plausible causes [135,136].

Therefore, when interpreting diagnostic findings, consideration should be given to the quality and reproducibility of the available evidence, its applicability to the specific clinical setting, and whether it supports interventions with demonstrated benefits for clinically meaningful reproductive outcomes. When the clinical utility of additional testing remains uncertain, patients should be informed about its limitations, costs, and potential risks before it is performed [137].

## 6. Pharmacological Optimization

In patients with RIF, pharmacological therapies are employed to target hormonal, immunological, and vascular pathways involved in implantation or to address potentially modifiable abnormalities in endometrial preparation. However, the strength of evidence varies considerably among these strategies [138,139,140]. While immunomodulatory and antithrombotic medications are indicated only in specific clinical contexts, optimizing estrogen and progesterone exposure is a well-established part of cycle preparation. Therefore, rather than being administered empirically to all patients with RIF, treatment should be guided by a clearly defined and clinically actionable abnormality [141].

### 6.1. Progesterone

The primary hormone that controls decidualization, secretory transformation, and the development of endometrial receptivity is progesterone. Because endogenous progesterone synthesis is either absent or limited in programmed frozen embryo transfer cycles, exogenous supplementation is necessary for implantation and early pregnancy. Therefore, one potentially modifiable factor contributing to implantation failure is inadequate progesterone exposure [120,142].

The absorption, metabolism, and circulating concentrations achieved with vaginal progesterone vary considerably among individuals. Poorer reproductive outcomes in programmed cycles have been linked to low serum progesterone concentrations around the time of embryo transfer, according to observational data. Reported thresholds associated with poorer reproductive outcomes in FET cycles generally range from approximately 8 to 11 ng/mL, although no universally accepted cut-off has been established [119]. In the multicenter prospective ProFET study, serum progesterone concentrations <7.8 ng/mL on the day of FET were associated with a lower live birth rate than concentrations ≥7.8 ng/mL (28.2% vs. 40.0%; aOR 0.41, 95% CI 0.18–0.91; *p* = 0.028) [119]. However, the proposed thresholds vary according to the progesterone formulation, route of administration, timing of measurement, and assay used. Furthermore, due to the first uterine-pass effect, serum concentrations after vaginal administration may not accurately reflect endometrial tissue exposure [119,143].

Progesterone resistance should be distinguished from insufficient exposure to progesterone. Modifying the supplementation regimen may correct inadequate progesterone exposure resulting from insufficient hormone availability due to dose, route of administration, timing, absorption, metabolism, or adherence [88,144]. In contrast, progesterone resistance refers to reduced endometrial responsiveness despite apparently adequate progesterone exposure and may result from alterations in progesterone receptor expression, receptor co-regulators, epigenetic regulation, or downstream decidualization pathways. Progesterone resistance cannot currently be reliably identified using a validated clinical test, and no proven therapy is available to specifically overcome this condition [88,145].

Vaginal micronized progesterone, subcutaneous progesterone, intramuscular progesterone in oil, and oral progestogens like dydrogesterone are among the available formulations. While subcutaneous and intramuscular methods often result in more consistent blood concentrations, vaginal treatment offers substantial local uterine exposure. Individualized rescue strategies, including increasing the progesterone dose or adding a parenteral route in patients with low circulating progesterone concentrations, have shown promising results in observational studies [146,147,148].

However, the optimal timing for progesterone measurement, the appropriate threshold for intervention, and the most effective rescue strategy remain to be established. Therefore, although low serum progesterone identifies a potentially modifiable factor in programmed frozen embryo transfer cycles, evidence that progesterone monitoring and individualized rescue strategies improve live birth rates remains less definitive. Progesterone monitoring should therefore be incorporated into a well-defined clinical protocol rather than used as an isolated test [121,149].

### 6.2. Estrogen

Progesterone-mediated secretory transformation of the tissue is facilitated by estrogen, which also promotes endometrial growth. Estradiol can be given orally, transdermally, or vaginally during programmed frozen embryo transfer cycles; the route of administration depends on individual response, tolerability, and clinical protocol [36,150].

Although endometrial thickness is an imperfect marker of functional receptivity, persistently inadequate endometrial development remains a therapeutic challenge and has been associated with lower implantation and pregnancy rates. In cases of inadequate endometrial development, clinicians may extend the duration of estrogen therapy or adjust its dose or route of administration. Excessive therapy may prolong the cycle without addressing the underlying mechanism, and there is no evidence that progressively increasing estrogen exposure improves live birth rates [151,152].

Potential contributory factors, such as intracavitary adhesions, prior endometrial damage, altered uterine perfusion, and poor treatment adherence should be considered. Therefore, estrogen optimization should not be considered a therapy for all types of endometrial dysfunction or progesterone resistance; rather, it is most useful when inadequate proliferative development has been established [153,154].

### 6.3. Corticosteroids

Since corticosteroids can suppress the release of pro-inflammatory cytokines and modulate innate and adaptive immune cells, these drugs have been suggested as immunomodulators. Prednisolone is the corticosteroid most commonly investigated in reproductive medicine, particularly in women with suspected increased NK-cell activity or abnormal cytokine profiles [141,155,156].

However, despite this rationale, randomized trials have not demonstrated consistent benefits of corticosteroids on implantation, ongoing pregnancy, or live birth rates in general IVF or RIF populations. In a multicenter, double-blind, placebo-controlled randomized trial of 715 women with RIF, live birth occurred in 37.8% (135/357) of women receiving prednisone compared with 38.8% (139/358) receiving placebo (RR 0.97, 95% CI 0.81–1.17; *p* = 0.78) [157]. Interpretation of the available evidence is further complicated by inconsistent definitions of immune dysregulation and the lack of validated diagnostic tests to identify patients most likely to benefit from corticosteroid therapy. Abnormal peripheral NK cell counts and/or cytokine profiles should not be considered indications for treatment in isolation [157,158].

Current evidence does not support the routine empirical use of corticosteroids for RIF, including in patients selected solely on the basis of unvalidated immune abnormalities. In patients with autoimmune or inflammatory disorders requiring corticosteroid therapy for indications unrelated to RIF, treatment should follow disease-specific clinical guidelines and should not be regarded as therapy for RIF. Potential systemic adverse effects, including glucose intolerance, hypertension, and infections, should also be considered [141,156].

### 6.4. Antithrombotic Therapy

Low-dose aspirin and low-molecular-weight heparin (LMWH) have been investigated as potential therapies for implantation failure because of their proposed effects on coagulation, inflammation, the complement system, trophoblast function, and vascular biology. Although these mechanisms provide a biological rationale for treatment, they do not demonstrate that antithrombotic therapy improves implantation outcomes in patients without thrombotic or autoimmune disorders [159].

The clearest indication for antithrombotic therapy is confirmed antiphospholipid syndrome (APS), particularly in patients who meet the clinical and laboratory criteria for obstetric APS. However, even in this population, the available evidence primarily supports its use for preventing pregnancy loss and placental complications rather than improving implantation. There is insufficient evidence to support treatment with aspirin or LMWH for RIF alone, inherited thrombophilia without a relevant clinical history, or impaired uterine perfusion [160,161].

The use of aspirin or LMWH in all patients with RIF is therefore not indicated. Therapy should be considered only where there is an established maternal indication, with appropriate consideration of the risks of bleeding, bruising, thrombocytopenia, and other adverse effects [162,163].

### 6.5. Principles of Integrated Pharmacological Management

Current therapeutic approaches for RIF include combinations of hormonal, immunomodulatory, and anticoagulant therapies. Nevertheless, the use of multiple therapies without clear indications makes it difficult to determine the contribution of individual interventions while increasing costs, adverse effects, and treatment burden. Interpretation of the literature is further limited by inconsistent definitions of RIF, heterogeneous treatment protocols, small sample sizes, and the frequent use of implantation or clinical pregnancy rather than live birth as primary outcomes [139,140,153].

The most reasonable approach is to optimize established endometrial preparation and reserve pharmacological therapy for patients with clearly defined abnormalities and evidence-based indications. Inadequate progesterone exposure is one such potentially modifiable factor that may warrant adjustment of luteal phase support. On the other hand, immunological, thrombophilia, and vascular tests are insufficient reasons for the use of corticosteroids and anticoagulants. There is no standardized pharmacological therapy for women with RIF at present [116,164,165].

The principal pharmacological interventions, their proposed mechanisms, populations and potential indications, comparators, reproductive outcomes, qualitative certainty of evidence, and current clinical position are summarized in Table 3.

## 7. Emerging and Investigational Therapies

Interventions aimed at enhancing endometrial development or receptivity through regenerative, angiogenic, immunologic, or microbiome-based approaches have been proposed. The most commonly studied interventions include PRP, G-CSF, and microbiome-targeted therapies [166]. Despite promising findings from early studies, the current evidence remains weak because of heterogeneity in patient populations, definitions of RIF and thin endometrium, treatment protocols, and reliance on surrogate rather than clinically meaningful outcomes. None of these interventions can currently be considered standard care for patients with RIF [167,168].

### 7.1. Platelet-Rich Plasma

Platelet-rich plasma refers to an autologous blood preparation with a higher platelet concentration than that of peripheral blood. Upon activation, platelets release a variety of bioactive molecules, including platelet-derived growth factor, transforming growth factor-β, vascular endothelial growth factor, insulin-like growth factor-1, epidermal growth factor, and basic fibroblast growth factor. These mediators promote angiogenesis, extracellular matrix remodeling, cell proliferation, and tissue regeneration [169,170].

PRP has been investigated primarily in women with refractory thin endometrium and, less frequently, in patients with RIF. Some observational studies and clinical trials have reported improvements in endometrial thickness and potential increases in implantation and pregnancy rates following intrauterine PRP administration. Interpretation and comparison of the available evidence are complicated by heterogeneity in blood processing methods, platelet concentration, leukocyte content, platelet activation, PRP volume, number of injections, and other methodological factors [171].

Although some randomized trials and meta-analyses have suggested beneficial effects on reproductive outcomes, the overall quality of the available evidence remains low. Several studies are limited by small sample sizes, an unclear risk of bias, and the use of clinical pregnancy rather than live birth as the primary outcome. Moreover, improvements in endometrial thickness do not necessarily translate into higher live birth rates. Although PRP is an autologous product and appears to be associated with few adverse effects, long-term safety and follow-up data remain limited [168,169,170]. Therefore, the available evidence is insufficient to support routine PRP use for RIF, and its application should remain investigational pending adequately powered trials evaluating live birth outcomes.

### 7.2. Granulocyte Colony-Stimulating Factor

Granulocyte colony-stimulating factor (G-CSF) is a cytokine that regulates neutrophil production and function and has also been proposed to promote cellular proliferation, angiogenesis, immune modulation, and the recruitment of bone marrow-derived progenitor cells. The identification of G-CSF receptors on endometrial epithelial and stromal cells provides a biological rationale for a local role of this cytokine in endometrial development and receptivity [172,173].

Studies on G-CSF have investigated both intrauterine and systemic routes of administration and have mainly focused on women with refractory thin endometrium and RIF. Although some observational studies and clinical trials have reported improvements in endometrial thickness, implantation rates, and clinical pregnancy rates, randomized trials have yielded inconsistent results. Interpretation of the evidence is further complicated by heterogeneity in routes of administration, dosing regimens, treatment frequency, and patient selection criteria [174,175,176].

Based on the available evidence, it cannot be concluded that G-CSF therapy improves live birth rates or that any potential benefit is confined to a reliably identifiable patient subgroup. Potential adverse effects, including bone pain, transient leukocytosis, and headache, should also be considered. Therefore, current evidence is insufficient to support the routine use of G-CSF for RIF or refractory thin endometrium, and its use should remain investigational pending the results of high-quality randomized controlled trials [174,175,176].

### 7.3. Microbiome-Directed Interventions

The role of the reproductive tract microbiota in implantation has raised the possibility of therapies that modify its composition. Proposed therapeutic strategies include antibiotics, probiotics, prebiotics, or combinations thereof to increase the abundance of *Lactobacillus* species or reduce potentially pathogenic microorganisms [177,178].

Some studies have associated a *Lactobacillus*-predominant endometrial microbiota with improved reproductive outcomes, whereas non-*Lactobacillus*-predominant microbiota have been associated with implantation failure [177,178]. Nevertheless, these findings do not establish a causal relationship or demonstrate that modifying the endometrial microbiota improves implantation. Interpretation of the available evidence is further complicated by the low microbial biomass of endometrial samples, the risk of contamination, differences in sampling sites, and substantial methodological heterogeneity in sequencing techniques and bioinformatic analyses [177].

Empirical microbiota modification should be distinguished from the treatment of chronic endometritis supported by histological or clinical evidence. When chronic endometritis is diagnosed, antibiotic therapy may be considered [74,122]. However, there is insufficient evidence to support the use of antimicrobial therapy solely to modify a sequencing-based endometrial microbial profile. Additionally, unnecessary antibiotic exposure may disrupt the commensal microbiota, with potential consequences including antimicrobial resistance [179,180].

Evidence supporting probiotics or other microbiome-restoration strategies remains limited, and it has not been established that vaginal microbial alterations consistently correspond to changes in the endometrial microbiota or that their modification improves live birth rates. Therefore, microbiome-directed therapies should be considered investigational and should not be recommended solely on the basis of an unvalidated finding of dysbiosis [127,181].

### 7.4. Methodological Considerations and Research Priorities

The interpretation of the evidence relating to the use of PRP, G-CSF, and microbiome-directed therapies is limited by several methodological issues, including small sample sizes, inconsistent definitions of RIF and thin endometrium across published studies, differences in embryo quality, varied treatment protocols, lack of blinding, short follow-up and selective reporting of surrogate endpoints. Furthermore, improvements in endometrial thickness, microbiome composition, implantation rates, or clinical pregnancy rates should not be interpreted as evidence of meaningful clinical benefit unless they ultimately translate into higher live birth rates [168,169,174].

Future clinical trials will require standardized methods for treatment preparation and administration, clearly defined and mechanistically plausible eligibility criteria, and relevant comparator treatments. Stratification according to embryo ploidy status, patient phenotype, and previous treatment history would facilitate interpretation of the results. Primary outcome measures in future trials should include live birth rate, time to pregnancy, and maternal and neonatal adverse outcomes rather than being limited to surrogate endpoints [4,168,169].

Currently, PRP, G-CSF, and microbiome-directed therapies should be regarded as investigational rather than standard adjunctive treatments for RIF. When used outside the context of clinical research, patients should be informed about the uncertainty surrounding their efficacy, as well as their costs, invasiveness, and lack of established long-term benefit [168].

## 8. Controversial and Empirical Approaches

Several treatments used in reproductive medicine illustrate the discrepancy between biological plausibility and proven clinical efficacy. Endometrial scratching, intralipid therapy, intravenous immunoglobulin, and intrauterine administration of human chorionic gonadotropin were adopted into clinical practice based on promising early scientific evidence. However, subsequent randomized trials and systematic reviews have not convincingly demonstrated an improvement in live birth rates. Their continued use highlights the importance of distinguishing biological rationale from proven clinical benefit [4,168].

### 8.1. Endometrial Scratching

The concept of endometrial scratching is based on deliberate physical injury to the endometrial lining and is typically performed in a cycle preceding embryo transfer. The proposed mechanism is that procedure-induced inflammation recruits immune cells to the site of injury, thereby promoting tissue remodeling and enhancing endometrial receptivity in the subsequent cycle [182].

Subsequent larger, well-designed randomized trials have failed to demonstrate a clinical benefit in unselected IVF populations. In a multicenter randomized trial of 1364 women undergoing IVF, live birth occurred in 26.1% (180/690) of women undergoing endometrial scratching and 26.1% (176/674) of controls (adjusted OR 1.00, 95% CI 0.78–1.27). No benefit was identified in the subgroup of women with at least two previous unsuccessful embryo transfers [182]. Systematic reviews have likewise shown no improvement in live birth rates. However, studies focusing specifically on patients with RIF have been difficult to interpret because of heterogeneity in the definition of RIF and the treatment protocols [4,182]. Furthermore, no reproducible subgroup of patients has been identified that consistently benefits from this intervention.

Thus, endometrial scratching should not be offered routinely to women with RIF. Given the absence of demonstrated clinical benefit, its invasiveness, discomfort, inconvenience, and potential cost further argue against routine use. Any further studies of this method should be conducted in adequately powered trials using a clear definition of RIF and live birth as a primary outcome [4,182].

### 8.2. Intralipid Therapy

Intralipid is a lipid emulsion developed for parenteral nutrition but has been used empirically in reproductive medicine based on findings from laboratory studies suggesting that it may reduce NK cell cytotoxicity and modulate cytokine secretion and immune cell membrane function [140,183].

Clinical evidence remains limited and methodologically heterogeneous. Differences in inclusion criteria, immunological assays, intravenous infusion protocols, concomitant interventions, and reproductive outcome measures preclude meaningful comparisons across studies. Intralipid administration is often accompanied by corticosteroids, aspirin, LMWH, and other concomitant therapies; therefore, it is difficult to attribute observed outcomes specifically to intralipid. Randomized evidence has not demonstrated a consistent live birth benefit in women with RIF [183].

There is currently no validated biomarker that can predict which patients are most likely to benefit from intralipid infusion. Therefore, intralipid should not be used routinely, and abnormal peripheral NK cell levels should not be considered a valid indication for its use. In the absence of validated patient-selection criteria and evidence of clinical benefit, intralipid should be limited to well-designed clinical trials and should not be considered an established component of mechanism-informed management for RIF [4,140].

### 8.3. Intravenous Immunoglobulin

Intravenous immunoglobulin (IVIG) exerts multiple immunomodulatory effects, including modulation of Fc receptor signaling, complement activation, antibody neutralization, cytokine signaling, and the activity of innate and adaptive immune cells. Based on these properties, IVIG has been investigated in patients with presumed immune-mediated implantation failure [140,184].

Current evidence does not consistently support improvements in live birth, ongoing pregnancy, or implantation rates in patients with RIF. Small sample sizes, inconsistent definitions of immunological dysfunction, variable dosing schedules, and the frequent use of concomitant treatments limit the interpretation of the available evidence. Furthermore, the subgroup of patients with RIF most likely to benefit from IVIG cannot currently be reliably identified using established diagnostic tools [140,184].

IVIG is expensive, requires intravenous administration, and can result in aseptic meningitis, thromboembolic events, headaches, hemolysis, infusion reactions, and renal failure. Although current manufacturing processes have substantially reduced the risk of pathogen transmission, the overall treatment burden and potential serious adverse effects remain important considerations. IVIG should be reserved for approved indications or evaluated in well-designed clinical trials; its routine use for RIF is not recommended [4,184].

### 8.4. Intrauterine hCG Lavage

Intrauterine administration of human chorionic gonadotropin (hCG) prior to embryo transfer has been proposed based on luteinizing hormone/human chorionic gonadotropin receptor expression in endometrial tissue and the role of embryo-derived hCG in embryo–endometrial dialogue. The proposed effects include modulation of cytokine signaling, immune cell recruitment, angiogenesis, and trophoblast attachment [30,44,106].

Results from randomized trials and meta-analyses have been mixed. Some studies involving cleavage-stage embryo transfers have reported improvements in implantation or clinical pregnancy, whereas studies involving blastocyst transfers have not demonstrated significant benefit. Variation in embryo stage, hCG dose, timing, and route of administration, and study quality further complicates the interpretation of the available evidence. Moreover, there is no clear evidence of improved live birth rates [185,186].

There is currently insufficient evidence to support the routine use of intrauterine hCG administration prior to embryo transfer, including in patients with RIF. The benefits reported in some studies involving cleavage-stage embryo transfer have not been consistently reproduced across embryo stages or translated into convincing live birth benefit and therefore do not justify routine clinical use [4,168].

The evidence supporting the investigational and empirical interventions discussed in Section 7 and Section 8, including the populations evaluated, comparators, reproductive outcomes, certainty of evidence, and current clinical position, is summarized in Table 4.

### 8.5. Common Evidence Pattern and Clinical Implications

These interventions illustrate the tendency in reproductive medicine for rapid clinical adoption to be driven by biological plausibility and promising early observational data, even in the absence of evidence of efficacy from adequately powered randomized trials. In small studies in particular, the risk of selection bias, inconsistent definitions of RIF, embryo heterogeneity, confounding effects of concomitant therapies, and publication bias toward positive results is high [108,132,137,168].

The heterogeneity of RIF may mask a true treatment effect in a subgroup of responsive patients. Nonetheless, this possibility should not be used to justify the routine use of a treatment when the responsive phenotype has not yet been established. Individualized therapy requires a validated biomarker, a plausible treatment–biomarker interaction, and evidence of improved clinical outcomes [4,83,166,168].

When patients inquire about empirical therapies, the biological rationale for their use should be clearly distinguished from evidence supporting their clinical efficacy. Consideration should be given not only to cost but also to potential complications and the adverse psychological impact associated with introducing multiple treatments [4,132,166]. Empirical interventions that have not demonstrated a benefit in terms of live birth rates should be used only in the context of clinical research [4,108,168].

## 9. Hypothesis and Discussion

### 9.1. Proposed Mechanistic Model

RIF may be conceptualized as a shared clinical endpoint resulting from disruption of one or more processes necessary for endometrial receptivity and embryo–endometrium communication when implantation failure continues after embryonic, laboratory, and procedural factors have been suitably assessed. Anatomical, inflammatory, temporal, hormonal, immunological, and vascular abnormalities may not always indicate distinct illnesses in this model. Instead, they represent overlapping mechanistic pathways that may contribute to impaired implantation [4,16,30,56].

This framework argues against a uniform diagnostic or therapeutic approach for every patient who meets the criteria for RIF. For instance, a cavity-distorting lesion or insufficient progesterone exposure would not be expected to be corrected by an intervention aimed at chronic endometritis. The therapeutic effect observed in a specific subgroup may be diluted when heterogeneous patient populations are analyzed without mechanistic stratification, as patients lacking the corresponding abnormality are included. Conversely, subgroup effects derived from small or post hoc analyses may be spurious and require prospective validation [4,56,108,168].

The boundaries between these proposed categories are not absolute. Progesterone resistance, inflammation, altered decidualization, and immunological dysfunction can all be associated with endometriosis or adenomyosis, whereas alterations in the local microbial composition and immune signaling may coexist with chronic endometritis. These connections do not imply that every patient has several issues or requires a combination therapy; rather, they support an integrated interpretation of the clinical findings within the broader mechanistic context [57,67,69,73].

### 9.2. Stratified Clinical Reasoning

Clinical decision-making within the framework proposed in this review is centered on the patient and the plausibility of the underlying endometrial mechanism. Mechanism-informed management refers to the selection or optimization of diagnostic and therapeutic strategies according to an identified or biologically plausible endometrial abnormality, whereas empirical add-on therapy refers to interventions applied without a validated diagnostic target or established clinical indication. Biological plausibility alone does not establish clinical utility, and investigational approaches should not be considered established components of mechanism-informed management until their diagnostic indications and therapeutic efficacy have been adequately validated. The goal is to identify abnormalities that are reproducible, physiologically plausible, and clinically actionable after embryonic, laboratory, procedural, and general maternal factors have been reviewed. Investigations should be prioritized according to the patient’s medical history, previous treatment outcomes, and the quality of evidence supporting both the diagnostic test and the corresponding intervention [4,42,56].

Within this framework, diagnostic findings should inform treatment prioritizations rather than serve as discrete labels. A positive result should influence management only when the test has adequate analytical and clinical validity and there is evidence that treating the discovered aberration is helpful. This distinction is especially crucial for vascular measures, immunological tests, microbiome profiling, and transcriptome receptivity testing, where biological signals may be detected without proven therapeutic usefulness [4,108,186].

Extensive testing or the accumulation of several supplementary therapies should not be equated with a mechanism-informed strategy. Rather, a mechanism-informed approach seeks to correct clinically relevant abnormalities, prioritizes validated and clinically supported diagnostic tests, and avoids treatments based solely on theoretical plausibility. Identification of a clinically relevant abnormality may establish a reasonable therapeutic priority; additional investigations should be pursued only when the overall clinical context supports another plausible mechanism [4,108,168].

**Figure 1:**
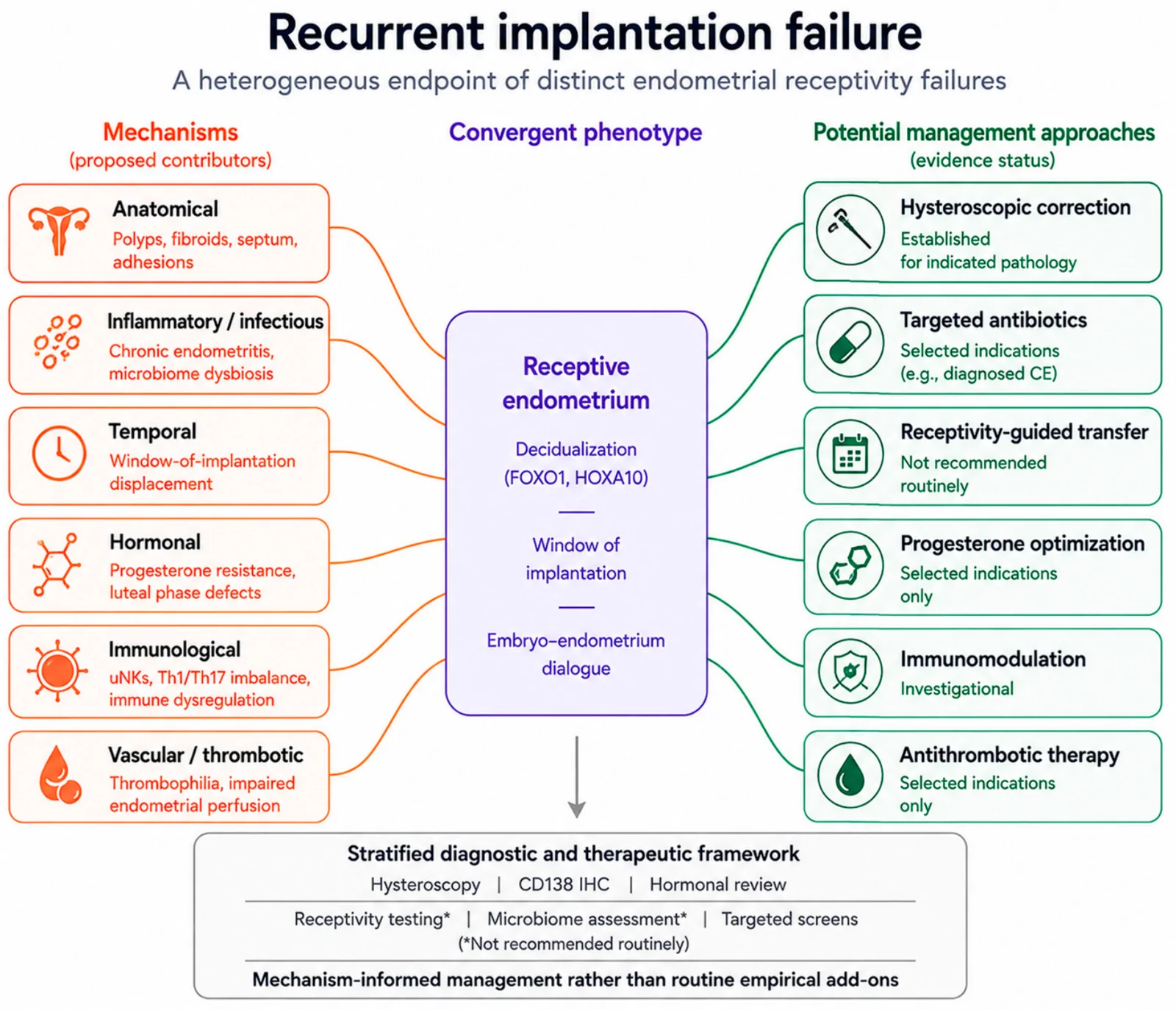
Mechanism-informed framework for endometrial factors contributing to recurrent implantation failure (RIF). Distinct endometrial mechanisms may converge on impaired receptivity and inform diagnostic and therapeutic considerations. Management approaches are categorized according to their current evidence status and should not be interpreted as uniformly established treatments. Abbreviations: CE, chronic endometritis; CD138, cluster of differentiation 138; FOXO1, forkhead box O1; HOXA10, homeobox A10; IHC, immunohistochemistry; RIF, recurrent implantation failure; Th, T-helper; uNKs, uterine natural killer cells.

Figure 1 depicts the proposed mechanism-informed framework linking potential endometrial abnormalities with their diagnostic evaluation and corresponding evidence-based or investigational management strategies.

### 9.3. Limits of Current Knowledge

There are several caveats that limit the precision of this model and its immediate clinical applicability. First, many of the reported endometrial abnormalities may merely represent correlations, transient physiological changes, or consequences of previous therapies rather than true pathogenic mechanisms, making it difficult to determine whether they contribute causally to implantation failure [4,56,167].

Second, the validity and clinical utility of diagnostic approaches vary considerably across mechanistic categories. Transcriptomic receptivity tests, peripheral and endometrial immune markers, microbiota analyses, and perfusion measures lack standardized cut-off values and evidence that their use improves clinical outcomes, whereas some uterine cavity abnormalities and suboptimal hormone levels can be identified using established clinical evaluation methods. Although progesterone resistance is biologically plausible and has been extensively investigated in conditions such as endometriosis, there is currently no validated method to diagnose endometrial progesterone resistance or predict the response to targeted intervention [4,88,108,186].

Third, heterogeneity in the definitions of RIF used across published studies, variability in embryo characteristics, small sample sizes, heterogeneous treatment protocols, and the frequent use of implantation or pregnancy rather than live birth as an outcome limit the interpretation of therapeutic studies. Evidence regarding the efficacy of sequential or combination treatments is particularly limited. Therefore, the possibility that multiple mechanisms may coexist should not be used to justify the empirical combination of several interventions [4,83,108,168].

Embryo–endometrium communication also remains incompletely understood. Most current research has examined the embryo and endometrium separately, limiting our understanding of the dynamic interactions that occur during implantation. RIF and recurrent pregnancy loss are distinct clinical entities, operationally differentiated by the presence or absence of an established clinical pregnancy, but may involve partially overlapping mechanisms, including abnormalities in decidualization, immune regulation, inflammation, and vascular remodeling. The extent of this mechanistic overlap remains uncertain and should not be assumed from shared molecular or cellular abnormalities alone [16,30,43,56].

Finally, rather than constituting a validated framework for clinical application, this approach should be regarded as a conceptual framework for identifying knowledge gaps and generating hypotheses that require prospective validation [4,56,168].

### 9.4. Implications for Clinical Trial Design

The efficacy of a medication in well-defined, mechanistically characterized subgroups may be underestimated or remain undetected in trials involving heterogeneous RIF populations defined using varying criteria. Inclusion criteria for next-generation studies should be transparent and aligned with current evidence, taking into account patient age, number of previous embryo transfers, embryo stage, and, where available, embryo ploidy status. Before being used for patient stratification, candidate biomarkers should be prospectively defined and analytically validated [4,56,83,132].

The goal of biomarker-guided trials should be to determine whether biomarker status modifies the benefits and risks of treatment, that is, whether the effect of an intervention depends on the proposed biological mechanism. A benefit observed in the biomarker-positive group alone does not justify the clinical use of the biomarker, as it neither establishes a treatment-by-biomarker interaction nor demonstrates that the biomarker reliably identifies patients who derive differential benefit from the intervention. Prospectively designed biomarker-stratified trials are therefore preferable to post hoc subgroup analyses, which carry a substantial risk of false-positive findings [132,137,168].

The selection of outcome measures also requires careful consideration. Although biochemical pregnancy and implantation rates may provide mechanistic insight, they may not reflect the outcomes that matter most to patients. Cumulative live birth per patient, assessed over a predefined timeframe or number of treatment cycles, may represent the most clinically meaningful primary outcome, while time to pregnancy, pregnancy loss, adverse events, treatment burden, neonatal outcomes, and cost-effectiveness should also be reported [4,108,168].

Adaptive and platform trial designs may facilitate the concurrent evaluation of multiple mechanism-informed interventions; however, their potential advantages should not compromise rigorous patient selection, treatment allocation, or evidence-based clinical practice. These designs do not eliminate the need for robust characterization of the patient population and appropriate use of control groups. Ultimately, biomarker-guided management can only be justified if it results in superior clinically meaningful outcomes compared with evidence-based management that is not guided by the biomarker [4,108,132,137].

## 10. Future Perspectives

Future advances in RIF evaluation and treatment will depend on biomarkers that accurately assess endometrial function and predict responses to targeted therapies. Pharmacogenetic and molecular analyses of progesterone receptor signaling and the decidualization cascade may help identify patients with impaired progesterone responsiveness. Decidualization tests incorporating clinically applicable biomarkers, including FOXO1, HOXA10, HAND2, microRNAs, and metabolic biomarkers, may help differentiate inadequate progesterone exposure from endometrial progesterone resistance. Nevertheless, these biomarkers require analytical validation, standardized sampling protocols, and evidence that their use improves clinically meaningful reproductive outcomes [17,24,88,166].

Endometrial secretome analysis represents another potential approach to minimally invasive assessment of endometrial function. Proteins, metabolites, lipids, cytokines, and extracellular vesicles present in uterine fluid could serve as minimally invasive biomarkers for evaluating endometrial receptivity around the time of embryo transfer. Integrating proteomic, transcriptomic, metabolomic, and extracellular vesicle analyses would provide a more comprehensive understanding of embryo–endometrial communication than analyzing the embryo and endometrium separately. However, sampling techniques still need to be demonstrated to be reproducible, clinically safe, and compatible with embryo transfer [17,30,166,187].

Clinical, embryological, hormonal, imaging, and multi-omics data could be incorporated through machine learning and AI techniques. These models could provide individualized estimates of implantation probability, classify patients into biologically meaningful subgroups, and predict treatment response to mechanism-informed interventions. However, models developed from retrospective, single-center datasets are susceptible to overfitting, bias, and limited generalizability. Prospective studies should include external validation, transparent reporting of model limitations, clinically meaningful outputs, and evaluation of whether model-guided decision-making improves outcomes compared with usual care [15,132,137].

Integrating research on RIF and RPL may also help elucidate shared mechanisms of early reproductive failure, including impaired decidualization, inflammation, immune dysregulation, and defects in embryo–endometrium dialogue. Although RIF and RPL are distinct clinical entities, comparisons across different stages of reproductive failure may reveal shared biological mechanisms that would otherwise be overlooked when each condition is analyzed separately [16,27,56].

Ultimately, technological sophistication alone will not establish clinical value. Novel biomarkers, multi-omics signatures, and predictive models should be assessed according to their reproducibility, incremental clinical utility beyond established clinical parameters, ability to inform a specific clinical intervention, effects on cumulative live birth and patient safety, treatment burden, and cost-effectiveness [4,108,132,166].

## 11. Conclusions

RIF represents a heterogeneous clinical endpoint rather than a distinct nosological entity. Contributing factors include anatomical, inflammatory, temporal, hormonal, immunological, and vascular abnormalities. These factors may act through distinct but overlapping pathways; however, identifying an abnormality does not establish its causal role or clinical relevance. Consequently, no single diagnostic test or therapeutic strategy is appropriate for all women with RIF.

Management should first involve an individualized assessment of embryonic, maternal, laboratory, procedural, and endometrial factors, with correction of clinically relevant abnormalities that are reproducibly identified. Established diagnostic and management strategies should be preferred, and the use of unvalidated tests and empirical add-on therapies should be minimized or restricted to research settings. Future advances will depend on prospectively validated biomarkers and robust evidence supporting mechanism-informed management that improves cumulative live birth rates while ensuring patient safety and addressing patients’ reproductive goals.

## Figures and Tables

**Table 1:** Mechanistic classification of endometrial factors potentially involved in recurrent implantation failure, with corresponding diagnostic approaches, potential management strategies, and current clinical status. Abbreviations: APS, antiphospholipid syndrome; FET, frozen embryo transfer; HLA, human leukocyte antigen; LMWH, low-molecular-weight heparin; RIF, recurrent implantation failure; uNK, uterine natural killer.

Category	Principal Entities/Mechanisms	Potential Diagnostic Approach	Potential Management and Current Clinical Status	References
Anatomical	Submucosal fibroids; polyps; intrauterine adhesions; uterine septum; adenomyosis	TVUS; 3D US; SIS; hysteroscopy; MRI (selected cases)	Correct cavity-distorting lesions; individualized adenomyosis management	[60,63,65]
Inflammatory/infectious	Chronic endometritis; endometrial dysbiosis	Endometrial biopsy ± CD138; microbiology if indicated; microbiome profiling (investigational)	Treat confirmed CE; microbiome therapies investigational	[69,70,77]
Temporal	Altered window of implantation	Endometrial receptivity testing (selected cases/research)	Personalized ET remains controversial; not routine	[4,35,82]
Hormonal	Low progesterone exposure; progesterone resistance; impaired decidualization; inadequate endometrial development	Cycle review; serum progesterone; ultrasound; decidualization biomarkers (investigational)	Optimize progesterone/estrogen; no treatment for progesterone resistance	[24,27,88]
Immunological	uNK-cell abnormalities; cytokine dysregulation; Th1/Th2/Th17/Treg imbalance	No validated clinical test; immune testing investigational	Immunomodulation not routinely recommended	[16,49,93]
Vascular/thrombotic	APS; thrombophilia; microvascular dysfunction; impaired perfusion	APS testing; thrombophilia testing (selected patients); Doppler (investigational)	Treat APS; avoid empirical aspirin/LMWH in unselected RIF	[4,100,103]

**Table 2:** Proposed stepwise approach to the diagnostic evaluation of recurrent implantation failure and the current clinical status of available investigations. Abbreviations: APS, antiphospholipid syndrome; CD138, cluster of differentiation 138; ERA, Endometrial Receptivity Analysis; FET, frozen embryo transfer; HLA, human leukocyte antigen; IVF, in vitro fertilization; PGT-A, preimplantation genetic testing for aneuploidy; RIF, recurrent implantation failure; SIS, saline infusion sonography; uNK, uterine natural killer.

Tier	Investigation	Suggested Indication	Clinical Implication/Current Status	References
Initial assessment	Confirm RIF phenotype; review maternal, embryonic, laboratory and procedural factors	All suspected RIF	Excludes non-endometrial causes; identifies correctable factors	[4,56,133]
Initial assessment	Review cycle preparation, endometrial development, progesterone exposure and ET timing	All RIF patients	Optimizes IVF/FET protocol and luteal support	[4,32,119]
Established	TVUS ± 3D US	Persistent implantation failure	Assesses uterine cavity, endometrium, fibroids, adenomyosis and uterine anomalies	[111,112,114]
Clinically indicated	SIS or hysteroscopy	Suspected intracavitary pathology; abnormal imaging; previous uterine surgery	Confirms and may treat cavity abnormalities; routine hysteroscopy not recommended after normal imaging	[4,115,134]
Clinically indicated	Serum progesterone	Programmed FET; suspected low progesterone	Detects inadequate exposure; guides progesterone optimization	[119,120,121]
Selected	Endometrial biopsy + CD138	Suspected chronic endometritis	Confirms CE; may guide antibiotic therapy	[69,123,124]
Investigational	ERA or other receptivity tests	Persistent unexplained RIF (preferably research)	Personalized ET remains unproven; not routine	[4,35]
Investigational	Endometrial/reproductive tract microbiome	Research setting	No validated diagnostic thresholds or treatments	[75,77,127]
Clinically indicated	APS and targeted thrombophilia testing	Relevant clinical history	Guides evidence-based treatment; routine screening not recommended	[4,103]
Investigational	Endometrial immune assessment	Research setting	No validated tests or proven clinical utility	[16,93,129]
Investigational	HLA compatibility and advanced immune panels	Research setting	Clinical utility unproven	[4,49,129]
Investigational	Uterine/subendometrial Doppler	Research or selected patients	Clinical role remains uncertain	[50,130,131]

**Table 3:** Pharmacological interventions in RIF: clinical indications, reproductive outcomes, evidence, and current clinical position.

Intervention	Proposed Mechanism/Target	Population/Potential Indication	Comparator	Main Outcomes Evaluated	Evidence/Current Position	Certainty	References
Estrogen	Endometrial proliferation; progesterone receptor expression	Programmed FET; inadequate endometrial development	Alternative estrogen regimens/routes or standard endometrial preparation	Endometrial thickness and pregnancy outcomes; limited RIF-specific live-birth evidence	Established component of programmed FET; route and dose should be individualized according to endometrial response	Moderate	[18,32,150]
Progesterone	Secretory transformation; decidualization; endometrial receptivity	Luteal support; programmed FET; low serum progesterone	Standard luteal support or differing progesterone regimens	Serum progesterone, clinical/ongoing pregnancy, and live birth	Standard component of FET preparation; low progesterone is associated with poorer outcomes and may justify adjustment of dose, route, or timing	Moderate	[24,119,120]
Combined/parenteral progesterone	Increases systemic progesterone exposure	Low serum progesterone; suspected inadequate exposure during FET	Standard vaginal progesterone alone	Ongoing pregnancy and live birth	Rescue supplementation is supported by observational/prospective evidence, but thresholds and protocols remain heterogeneous	Low–moderate	[149,164,165]
Corticosteroids	Modulation of inflammatory and immune responses	RIF; proposed immune abnormalities; established autoimmune/inflammatory disease	Placebo, no corticosteroid, or standard care	Implantation, clinical pregnancy, and live birth	No demonstrated live-birth benefit in unselected RIF; routine empirical use is not recommended	Moderate	[4,141,157]
Low-dose aspirin	Antiplatelet and vascular effects	RIF; established cardiovascular or obstetric indications	Placebo/no aspirin or standard care	Implantation, pregnancy, and live birth	No convincing evidence of benefit for isolated RIF; use should be restricted to established indications	Low	[4,162,163]
Low-molecular-weight heparin	Anticoagulant effects; proposed effects on implantation	RIF; APS or other established anticoagulation indications	Placebo/no LMWH or standard care	Implantation, clinical pregnancy, and live birth	Evidence does not support empirical treatment of unselected RIF; recommended when an established anticoagulation indication exists	Low for isolated RIF	[4,161,163]

**Table 4:** Investigational and empirical interventions in RIF: populations studied, reproductive outcomes, evidence, and current clinical position.

Intervention	Main Population Evaluated	Comparator	Principal Outcomes	Overall Evidence	Narrative Certainty	Current Position	References
PRP	RIF and/or refractory thin endometrium	Placebo, no intervention, or standard endometrial preparation	Endometrial thickness, implantation, clinical pregnancy; some studies report live birth	Meta-analyses suggest possible benefit, but studies are small and heterogeneous in PRP preparation and administration	Low	Investigational; routine use not established	[168,169,170,171]
G-CSF	RIF and refractory thin endometrium	Placebo/no G-CSF/standard treatment	Endometrial thickness, implantation, clinical pregnancy; live-birth evidence inconsistent	Results from trials and meta-analyses are inconsistent; substantial protocol and population heterogeneity	Low	Investigational; routine use not recommended	[172,173,174,175,176]
Microbiome-directed therapies	Women with presumed endometrial/reproductive-tract dysbiosis or RIF	Standard care/no microbiome-directed intervention	Microbiome composition and pregnancy outcomes; robust live-birth evidence lacking	Associations between microbial profiles and outcomes exist, but intervention studies and validated dysbiosis thresholds are insufficient	Very low	Investigational; targeted treatment only for established indications such as confirmed chronic endometritis	[73,74,77,177,178,179,180,181]
Endometrial scratching	IVF patients, including subsets with previous implantation failure/RIF	Sham/no scratching	Live birth, clinical pregnancy, implantation	Large randomized trials have not demonstrated improved live birth; RIF-specific evidence remains heterogeneous	Moderate for lack of routine benefit	Routine use not recommended	[4,108,168,182]
Intralipid	RIF, often in women selected using immune/NK-cell abnormalities	Placebo/no intralipid or standard treatment; often with concomitant therapies	Implantation, clinical pregnancy and live birth	Small heterogeneous studies; no consistent randomized evidence of live-birth benefit	Very low	Not recommended routinely	[4,108,140,183]
IVIG	RIF, frequently presumed immune-mediated RIF	Placebo/no IVIG/standard treatment	Implantation, ongoing pregnancy and live birth	Meta-analytic signals are limited by small heterogeneous studies and poorly defined immune phenotypes	Very low	Routine use not recommended; clinical-trial setting preferred	[4,108,140,184]
Intrauterine hCG	Women undergoing embryo transfer, including previous implantation failure; cleavage-stage and blastocyst studies	Placebo/no intrauterine hCG	Implantation and clinical pregnancy; live-birth benefit not established	Findings differ according to embryo stage and protocol; no convincing live-birth advantage	Low	Routine use not recommended	[4,108,185,186]

## Data Availability

No new data were created or analyzed in this study.

## Appendix A

**Table A1:** Literature search strategies and results retrieved for the narrative review. Abbreviations: RIF, recurrent implantation failure; hCG, human chorionic gonadotropin. Search-result counts represent the number of records returned by each search at the time it was conducted. Because this study was designed as a narrative rather than a systematic review, records were not subjected to a formal deduplication or PRISMA-based screening procedure. Consequently, the reported counts should not be interpreted as numbers of unique records, and no aggregate total across sources is provided.

Database/Source	Search Strategy	Results Retrieved	Coverage
PubMed/MEDLINE	(“recurrent implantation failure” OR “implantation failure”) AND (“endometrial receptivity” OR “window of implantation” OR “decidualization” OR “progesterone resistance” OR “chronic endometritis” OR “endometrial microbiome” OR “uterine natural killer cells” OR “immune dysregulation” OR “vascular dysfunction” OR “thrombophilia” OR “endometrial receptivity testing” OR “personalized embryo transfer” OR “progesterone supplementation” OR “corticosteroids” OR “aspirin” OR “low-molecular-weight heparin” OR “platelet-rich plasma” OR “granulocyte colony-stimulating factor” OR “endometrial scratching” OR “intralipid” OR “intravenous immunoglobulin”)	1311	Inception to June 2026
Scopus	TITLE-ABS-KEY (“recurrent implantation failure” OR “implantation failure”) AND TITLE-ABS-KEY (“endometrial receptivity” OR “window of implantation” OR “decidualization” OR “progesterone resistance” OR “chronic endometritis” OR “endometrial microbiome” OR “uterine natural killer cells” OR “immune dysregulation” OR “vascular dysfunction” OR “thrombophilia” OR “endometrial receptivity testing” OR “personalized embryo transfer” OR “progesterone supplementation” OR “corticosteroids” OR “aspirin” OR “low-molecular-weight heparin” OR “platelet-rich plasma” OR “granulocyte colony-stimulating factor” OR “endometrial scratching” OR “intralipid” OR “intravenous immunoglobulin”)	1827	Inception to June 2026
ScienceDirect—Search 1: Endometrial receptivity	(“recurrent implantation failure” OR “implantation failure”) AND (“endometrial receptivity” OR “window of implantation” OR “decidualization” OR “progesterone resistance”)	2161	Inception to June 2026
ScienceDirect—Search 2: Inflammation, microbiome, and immunity	(“recurrent implantation failure” OR “implantation failure”) AND (“chronic endometritis” OR “endometrial microbiome” OR “uterine natural killer cells” OR “immune dysregulation”)	117	Inception to June 2026
ScienceDirect—Search 3: Vascular and coagulation factors	(“recurrent implantation failure” OR “implantation failure”) AND (“vascular dysfunction” OR “thrombophilia” OR “aspirin” OR “low-molecular-weight heparin”)	251	Inception to June 2026
ScienceDirect—Search 4: Receptivity testing and hormonal treatment	(“recurrent implantation failure” OR “implantation failure”) AND (“endometrial receptivity testing” OR “personalized embryo transfer” OR “progesterone supplementation” OR “corticosteroids”)	305	Inception to June 2026
ScienceDirect—Search 5: Regenerative and growth-factor therapies	(“recurrent implantation failure” OR “implantation failure”) AND (“platelet-rich plasma” OR “granulocyte colony-stimulating factor” OR “human chorionic gonadotropin” OR “hCG”)	362	Inception to June 2026
ScienceDirect—Search 6: Empirical and immunological add-ons	(“recurrent implantation failure” OR “implantation failure”) AND (“endometrial scratching” OR “intralipid” OR “intravenous immunoglobulin”)	1	Inception to June 2026
Cochrane Library	(“recurrent implantation failure” OR “implantation failure”) AND (“endometrial receptivity” OR “window of implantation” OR “decidualization” OR “progesterone resistance” OR “chronic endometritis” OR “endometrial microbiome” OR “uterine natural killer cells” OR “immune dysregulation” OR “vascular dysfunction” OR “thrombophilia” OR “endometrial receptivity testing” OR “personalized embryo transfer” OR “progesterone supplementation” OR “corticosteroids” OR “aspirin” OR “low-molecular-weight heparin” OR “platelet-rich plasma” OR “granulocyte colony-stimulating factor” OR “endometrial scratching” OR “intralipid” OR “intravenous immunoglobulin”)	2	Inception to June 2026
